# PD131 Literature Analysis And Hot Topics In Congenital Heart Disease Screening In China: A Bibliometrics Study Using CiteSpace

**DOI:** 10.1017/S0266462324003684

**Published:** 2025-01-07

**Authors:** Liu Liu, Yi Yang, Shimeng Liu, Yan Wei, Yingyao Chen

## Abstract

**Introduction:**

Congenital heart disease (CHD), the most common congenital malformation affecting fetuses and infants, poses a significant and rapidly emerging global challenge in children’s health. Prenatal and newborn screening are very important for preventing CHD. Therefore, this study aimed to analyze the status and corresponding foci of articles on CHD screening in the Chinese or English language using bibliometric methods.

**Methods:**

Publications on prenatal or newborn screening for CHD were included. The Web of Science Core Collection (WoS) and China National Knowledge Infrastructure (CNKI) databases were searched to identify literature published from inception to 31 March 2023. CiteSpace was used to perform bibliometric analysis on the number of publications, institutions, authors, and keywords to generate corresponding knowledge maps.

**Results:**

A total of 582 publications were retrieved from the WoS and 233 from the CNKI databases. There was an increasing trend in the number of English and Chinese articles published, with the trend beginning in 2010 for Chinese language articles and in 2007 for English language articles. In English language publications, GR Martin was the most influential author, and the top five institutions were from high-income countries. Among the Chinese language publications, Wenhong Ding was the most influential author and the Hunan Province Maternal and Child Health Care Hospital was the most commonly reported institution. Keyword analysis revealed that the most frequently occurring terms in both language publications were as follows: antenatal diagnosis, cardiac auscultation, and fetal echocardiography in English language articles and screening, prenatal screening, and fetal in Chinese language publications.

**Conclusions:**

Increasingly, articles on CHD screening have been listed in the WoS and CNKI databases. This bibliometric study provides the status and trends in the research on screening for CHD and may help researchers identify hot topics and explore new research directions in this field.

